# Application of Approximate Pattern Matching in Two Dimensional Spaces to Grid Layout for Biochemical Network Maps

**DOI:** 10.1371/journal.pone.0037739

**Published:** 2012-06-05

**Authors:** Kentaro Inoue, Shinichi Shimozono, Hideaki Yoshida, Hiroyuki Kurata

**Affiliations:** 1 Department of Bioscience and Bioinformatics, Kyushu Institute of Technology, Iizuka, Fukuoka, Japan; 2 Department of Artificial Intelligence, Kyushu Institute of Technology, Iizuka, Fukuoka, Japan; 3 Biomedical Informatics R&D Center, Kyushu Institute of Technology, Iizuka, Fukuoka, Japan; Université de Nantes, France

## Abstract

**Background:**

For visualizing large-scale biochemical network maps, it is important to calculate the coordinates of molecular nodes quickly and to enhance the understanding or traceability of them. The grid layout is effective in drawing compact, orderly, balanced network maps with node label spaces, but existing grid layout algorithms often require a high computational cost because they have to consider complicated positional constraints through the entire optimization process.

**Results:**

We propose a hybrid grid layout algorithm that consists of a non-grid, fast layout (preprocessor) algorithm and an approximate pattern matching algorithm that distributes the resultant preprocessed nodes on square grid points. To demonstrate the feasibility of the hybrid layout algorithm, it is characterized in terms of the calculation time, numbers of edge-edge and node-edge crossings, relative edge lengths, and F-measures. The proposed algorithm achieves outstanding performances compared with other existing grid layouts.

**Conclusions:**

Use of an approximate pattern matching algorithm quickly redistributes the laid-out nodes by fast, non-grid algorithms on the square grid points, while preserving the topological relationships among the nodes. The proposed algorithm is a novel use of the pattern matching, thereby providing a breakthrough for grid layout. This application program can be freely downloaded from http://www.cadlive.jp/hybridlayout/hybridlayout.html.

## Introduction

Rapid advances in molecular biology have revealed a detailed map for gene regulatory networks, signal transduction pathways and metabolic circuits. Visual representations of such networks are critically important to help researchers gain insight into a large-scale complex network [1], stimulating the interest in developing computational tools that support visualization of biochemical networks.

A scientific goal of drawing a comprehensive biochemical map is to facilitate human perception of its topological structure or understandings of how network pathways generate cellular functions. Obviously, it is tedious and laborious to make intuitive and heuristic layouts for large-scale complex networks. In general, an automatic drawing of such complex networks can be achieved by converting biochemical pathway data into a graph representation. Many types of drawing algorithms have been developed with their associated graphical notations [2]–[11]. Many visualization software programs have been presented to enhance the usability of drawings [12]–[16]. They focus on how to place objects and route their connections to render layouts in traditionally accepted styles [17]–[30].

Force-directed layout algorithms have widely been used for visualizing large-scale maps of biological networks [3], [31]–[33], including biological similarity relationship networks [34] and coarse-grained maps of protein-protein interactions [35]–[37]. Their basic idea is to model a graph as a mechanical system, where the nodes are repulsive particles and the edges are attractive interactions. A layout is determined when the forces drive the system to a steady state (a local minimum of energy). To find aesthetically pleasing drawings of network maps constraint-based layouts extends the force-directed approach with constraints on node position [38] or they uses simulated annealing algorithms or some heuristic algorithms to optimize a randomly generated initial placements [39]–[41]. An objective or cost function is defined to measure the quality of the layout and it should be optimized subject to given constraints on the objects in the network. In general, definition of a cost function is critical for human-understandable drawings. The constraints can include horizontal and vertical alignment of nodes [42], [43], non-overlapping nodes [44], [45], edge direction [23], closeness of grouped nodes [42], [43], orthogonal ordering between nodes [46], containment of nodes within clusters [47], [48], placement of nodes below other nodes in directed graphs [49], drawing cycles on a rectangle [50] and multilevel framework [51], [52]. Some algorithms require not only the topology of the network but also biological information such as subcellular localization [22]–[24], [42], [43] and biological process [48].

As alternative methods, the spectral analysis for graph visualization computes the layout of a graph using certain eigenvectors of related matrices [53]. It can compute global optimum efficiently and calculate fast, but the spectral method usually provides very heterogeneous layouts with high node density. Self-Organizing Maps (SOM) were employed to perform layout of directed graphs, either weighted or unweighted [54], [55]. It attempts to distribute the nodes uniformly within a topology (e.g., rectangle, sphere, heart shape), keeping nodes close to their neighbors.

It is practically important to avoid the overlapping of node areas and to attach a label with a molecular species name to each node for enhanced understanding or traceability of biochemical networks. While some algorithms such as the scan-line algorithm [45] have been presented to enforce non-overlap in single dimension, grid layout algorithms, which arrange the nodes of biochemical network maps to grid points, can be solutions to attach a label to each node without overlapping. Since we first proposed a grid layout algorithm in bioinformatics in 2005 [25], because all the nodes are arranged to geometrically aesthetic grid points to draw an orderly, balanced network map and the number of grid points (the map size) is arbitrarily determined to ensure a compact map. Especially, nodes on grid points are readable when zooming in a local region of large-scale maps. Our original grid layout algorithm (GL) converted biochemical network maps into graph representation and arranged their nodes to grid points so that a specifically designed cost function is minimized over all possible mappings [25]. This algorithm repeatedly updates the layout by moving nodes one by one according to the simulated annealing methods. LucidDraw (LD) adopted a similar cost function to GL [25], while it speeded up the layout process dramatically [56]. LD employed a neighborhood-test procedure that repeatedly tries to move every single node to its adjacent vacant site to lower down the cost function. This layout algorithm avoids a local minimum by the perturbation that moves each node to a randomly chosen neighboring location. To avoid a locally optimal layout, sweep calculation was presented [22], [24], where the costs changed by moving a node of interest are encoded, and then the cost differences corresponding to the movements are calculated by using the encoded data. Cerebral (CE) employed search-based layout algorithms, but used a stochastic approach to searching and an optimized scoring function to make the layout of large networks tractable [42], [43].

Despite those improvements, existing grid layouts that take account of the cost function regarding positional constraints through the entire optimization process remain to be improved in terms of calculation speed. In this paper, we challenge an alternative or novel approach to fast grid layout, which combines an approximate pattern matching algorithm with widely-used, fast, typical layout algorithms such as spectral analysis, force-directed algorithms and SOM. In this study, we focus on the grid layout algorithms that rely merely on network topology without any use of molecular component, process and function. Use of an approximate pattern matching algorithm redistributes the coarse layouts by such fast algorithms on the square grid points, while preserving the topological relationships among the nodes. The proposed algorithm is a novel use of the pattern matching, thereby achieving very fast grid layouts with good topological performances compared with other existing grid layout methods in our limited knowledge.

## Methods

### Hybrid Grid Layout Algorithm

**Figure 1:**
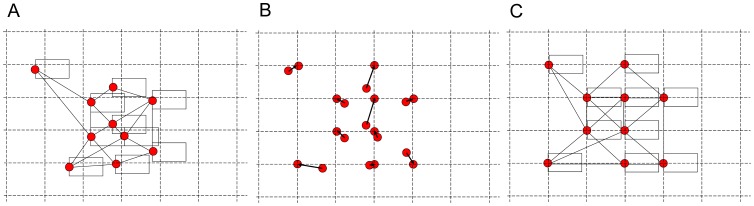
An image of the hybrid grid layout algorithm. A. An ordinary or preprocessor algorithm draws a network map where the node labels may overlap. B. An approximate pattern matching algorithm finds a one-to-one, exclusive correspondence between a set (pattern) of graph nodes and a set of the grid points with the minimum axis-parallel insertions and deletions of space. C. The spaces of text labels are ensured.

A biochemical network map can generally be converted into a graph to calculate the geometric coordinates of the molecules (nodes). The network graph consisting of *N* nodes and undirected edges (interactions) are described by an *N* by *N* adjacency matrix 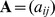
(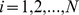, 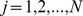). When there is an edge from a node *i* to another node *j*, then its element 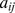 is 1, otherwise 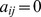. To draw the network map, it is necessary to calculate the x-y coordinates of *N* nodes 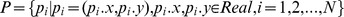. Practically, the text labels showing molecular names are critically important to trace the pathways of interest. It is important to secure the space necessary for node labels, but ordinary layout algorithms do not consider the label space. To obtain a view of a large-scale network graph whose nodes have text labels without any overlaps of them in a compact space, we propose the hybrid layout algorithm that maps molecular nodes on the grid points, while enhancing their topological quality, as shown in **Figure 1** and **Table 1**.

**Table 1:** An overview of hybrid grid layout algorithms.

Preprocessor algorithm
Fast, non-grid algorithms for determining the relative positions of network nodes *P*
Spectral Analysis
Kamada-Kawai algorithm
Fruchterman-Reingold algorithm
Gürsoy-Atun algorithm

Pattern matching
Pattern matching algorithm for distributing the preprocessed nodes to square grid points *G*
Divide and conquer method by the quad-tree: The layout area is divided into the subareas, where the node number is limited to “cut size”.
Depth-first recursive search for matching the preprocessed nodes on square grid points.

In the first stage, the positions of a set of graph nodes are roughly determined by typical, fast layout algorithms, named preprocessor algorithms. The preprocessor algorithms layout the nodes so as to improve topological features, such as a small number of edge-edge crossings, a short length of the total edges, and clear cluster structures, but they do not consider attaching text labels to nodes. In the second stage, an approximate point-set pattern matching algorithm finds a one-to-one and onto mapping from the graph nodes to grid points, i.e., assigns one grid point for each graph node exclusively. The computational problems to find an optimal point-set pattern matching on the plane defined so far is NP-hard [57]–[59]. Our algorithm is designed to minimize space insertion and deletions in a series of enhancement of axis-parallel bounding box of graph nodes, and runs in polynomial-time. The distance between the grid-lines secured by this second stage guarantees enough space for text labels of graph nodes. Furthermore, since the approximate pattern matching algorithm consumes up a large amount of memory space and takes a considerable amount of time, we propose, as a practical implementation, a divide-and-conquer strategy that can drastically reduce the computation time.

### Preprocessor Algorithms

There can be many candidates of preprocessor algorithms [60]–[64], but the investigation of all candidates are not practical. Thus, we selected four widely-used methods with different, typical algorithms.

#### Spectral analysis

Use of a spectral method minimizes the sum of squared edge length to calculate the coordinates of thousand nodes in a graph with a very fast speed, which considers its cluster structures. Clustering structures can be well represented or displayed by the spectral analysis, because elements with approximately equal values in the eigenvectors correspond to nodes having strong mutual connections [53]. The eigenvectors corresponding to small eigenvalues can be calculated in not more than 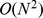 time, so that the spectral method is capable of clustering and drawing very large networks, e.g., a WWW network with 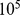 nodes. Nevertheless, the spectral method usually provides very heterogeneous layouts with high dense nodes and gives good results only in rather special networks. Thus, serious improvement is required to draw biochemical network maps that are understandable to humans.

#### Kamada-Kawai algorithm

KK [65] is a spring force directed layout algorithm, where the nodes are represented by steel rings and the edges are springs between them. The attractive force is analogous to the spring force and the repulsive force is analogous to the electrical force. The basic idea is to minimize the energy of the system by moving the nodes and changing the forces between them. They use the Newton-Raphson method for optimization with respect to a single node and reduce the overall stress by iteratively solving for each node. The time complexity is 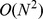 for each iteration of the algorithm in the worst case.

#### Fruchterman-Reingold algorithm

Fruchterman and Reingold [66] proposed a variant of Eades’ approach [67] as the spring force algorithm, in which attractive forces take into account the optimal distance between nodes, defined as a function of the number of nodes in the graph and the size of the drawing window. Differing from KK, this algorithm directly supports the layout of disconnected graphs and attractive forces occur between adjacent nodes only, whereas repulsive forces occur between every pair of nodes. Each iteration computes the sum of the forces on each node, and then moves the nodes to their new positions. Simulated annealing is used for optimization. The movement of nodes is mitigated by the temperature of the system. As the algorithm progresses through successive iterations, the temperature should decrease so that nodes settle in place. The cooling schedule, attractive forces, and repulsive forces can be provided by users. The time complexity is 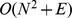 for each iteration of the algorithm in the worst case, where *E* is the number of edges.

#### Gürsoy-Atun algorithm

GA [54] performs layout of directed graphs, either weighted or unweighted. It employs an algorithm different from KK and FR, because it does not explicitly strive to layout graphs in a visually pleasing manner. Instead, it attempts to distribute the nodes uniformly within a topology (e.g., rectangle, sphere, heart shape), keeping nodes close to their neighbors. The algorithm is built based on Self-Organizing Maps.

### Pattern Matching Algorithm

#### Depth-first recursive search algorithm

As shown in **Table 1**, the nodes drawn by a preprocessor algorithm are aligned or matched to the grid points in a setting square space, while maintaining the relative positions of the preprocessed nodes. The pattern matching algorithm searches a pattern for the shortest movement from the preprocessed nodes to their grid points. The *N* preprocessed nodes drawn in a continuous space: 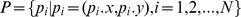, are mapped on the grid points: 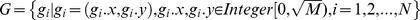, in the setting square space with *M* points one by one 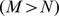. Overlaps of nodes are not allowed and all the permutations for *N* nodes are considered. When the *k-*th node is moved to its matched grid point, the other *k*+1 to *N-*th nodes that remain to be matched are moved in parallel together with the movement of the *k-*th matched node, where 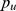 (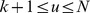) are updated as 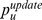, resulting in the temporal pattern:


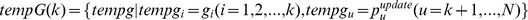
, where 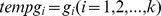 are on the grid points, indicated as integer vectors and 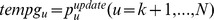 are given as real vectors. Once the nodes are matched, they are fixed. Without the parallel movement, it would take a long time to find the vacant grid point nearest to the preprocessed nodes, when many nodes are very condensed, i.e., the grid points close to the preprocessed node are readily full. Use of the parallel movement can save the search iterations because the nodes that have not matched yet move away from the condensed region.

The distance from a preprocessed node to its matched grid point is defined as Manhattan distance:



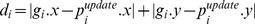



The sum of the matching distances for all *N* nodes is provided by:



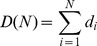



The matched pattern of grid points *G* is obtained by minimizing 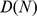.

As shown in **Table S1**, the depth-first recursive search algorithm is used to explore the layout pattern that minimizes 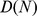, with respect to all the permutations of *N* nodes. The recursive algorithm stores the minimal 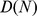 and the coordinates of the *N* matched grid points *G.* When newly calculated 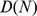 is less than the stored one, it and its associated coordinates of the grid points replace the stored ones; otherwise the stored ones are conserved. To save the calculation time, the sum of the *k*-node matching distance 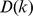
(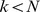) and the coordinates of its associated nodes 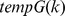 are stored. When 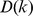 is more than the stored minimal 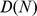, the further search from *k* to *N-*th nodes is omitted.

Matching of each node. Assuming that the nearest grid point to 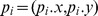 is vacant, the matched grid point 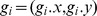 is provided by:


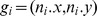
, 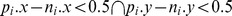.


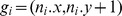
, 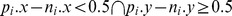.


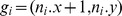
, 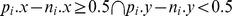.


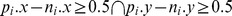
.

where 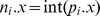, 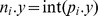. 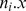 and 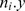 correspond to the integer parts of 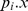 and 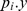, respectively. Actually, when the nearest node is full, it is necessary to search the nearest vacant grid point according to its distance from 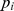. When (*k*-1) nodes are matched to the grid points, the nearest, vacant grid point for the *k*-th node is searched as shown in **Table S2** and **Figure S1**. The *k*-th grid point candidates 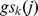 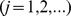 are sorted in the ascending order of Manhattan distance 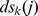 between 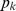 and 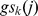. When 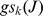 is the nearest vacant point, 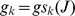 and 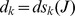.

#### Setting grid square space

The network map should be drawn within a compact space. In this study, we use 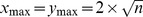 as the square, which is given by GL [25].

#### Algorithm’s complexity

Since it is necessary to store the sum of the *k*-node matching distance 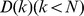 and the temporal pattern 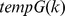 in each layer depth (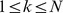), the space complexity is 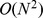. The time complexity requires 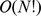 for all the permutations of nodes (NP-complete). Thus, we use the divide-and-conquer method to greatly reduce the calculation time.

### Divide-and-Conquer Strategy

Applying the recursion algorithm directly to a large set of nodes of a graph is difficult in practice, due to calculation complexity (**Figure S2**). The divide-and-conquer strategy is employed to accelerate the approximate pattern matching. We adopt the quad-tree [68] to divide the network into groups of the nodes and examine how the time complexity is balanced with the preference of resultant layouts. The quad-tree simply and recurrently divides the layout space into the quarters with the same area square until the number of nodes becomes less than until the partitioning parts contains a limited number of nodes, named cut size. We use the cut size of 10 for a high speed layout. This kind of strategy works very well if we can assume that points are in approximately uniform distribution.

### Combination of Preprocessor and Pattern Matching Algorithms

The four widely-used, fast layout algorithms are combined to the approximate pattern matching algorithm with the divide-conquer method. The resultant hybrid layout algorithms with spectral analysis, Kamada-Kawai algorithm, Fruchterman-Reingold algorithm, and Gürsoy-Atun algorithm are named SA, KK, FR, and GA, respectively.

### Reference Algorithms for Grid Layout

Four algorithms for random layout (R), GL, LD, and CE are used as reference or control methods. We selected the reference grid layout algorithms that rely merely on topological information without any biological constrains (molecular component, process, function).

#### Random layout (R)

Nodes are randomly scattered in a given grid space without any overlaps. Uniform random numbers are employed.

#### Our original grid layout algorithm (GL)

GL is an optimization algorithm for minimizing the cost function for layouts of networks [25]. The network is built as a system of interacting particles which are placed on a two-dimensional square grid and is confined within its area. The particles (nodes) interact according to a predefined energy function based on the network topological structure, where all edges are straight lines. The energy of the configuration of particles is the cost function of the corresponding layout. A stable configuration has low energy; equivalently, an acceptable layout has a low cost function.

#### LucidDraw (LD)

A good layout algorithm [56] depends on two factors: a proper cost function and an efficient optimization method. LD adopts a similar cost function as GL [25], while it speeds up the layout process dramatically, serving as an instant visualization tool in the context of a wide range of network analysis tasks. To reduce the search area of every node, the neighborhood-test method is used, greatly decreasing the computational cost. To fully optimize the cost function, the re-optimization-after-perturbation strategy is used to force the layout to escape from current local minimum and search for better layouts. The perturbation strategy, despite its simplicity, achieves rather good performance comparing to other sophisticated heuristics like simulated annealing. The technique was employed in other discrete global optimization problems [69], [70].

#### Cerebral (CE)

CE [42], [43] evaluates the quality of the node in its new position depending on a node’s function. Node distributions are evaluated based on edge length, node-edge crossings and edge-edge crossings. CE uses simulated annealing to search a minimum cost under the hard constraints (layout space) and soft constraints (energy function). CE divides the layout space on the *y* axis into regions sized proportionally to the number of nodes in each layer. The energy function is defined by edge length, edge-edge and node-edge crossings, and known biological function grouping. This is similar to the cost function defined by CBS-grid (Kojima et al. 2008). The time complexity of CBS-grid requires 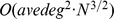, which *avedeg* is the average degree and *N* is the number of nodes, while that of Cerebral requires 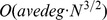. Cerebral is better than CBS-grid for the time complexity. Cerebral runs in the expected time 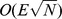 (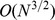 in the worst case) while using 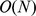 memory, where *E* is the number of edges.

### Measures for Characterizing Layouts

#### Calculation speed

The calculation time is measured to characterize calculation complexity for each layout algorithm. A personal computer (OS: Windows XP 32bit, CPU: Intel Core2Duo 3.0GHz, Memory: 3.2GByte) is used.

#### Edge-edge crossings

One measure of a graph drawing algorithm's quality is the number of edge-edge crossings it draws [22], [23], [42], [43]. Most graphs cannot be drawn without edge-edge crossings. According to this metric, good algorithms draw graphs with as few edge-edge crossings as possible. The ratio of edge-edge crossings is defined as the ratio of the number of edge-edge crossings to the total number of edge combinations.

#### Node-edge crossings

The problem of node-edge crossings should be avoided for biochemical network layout, because the node-edge crossings cause confusion where edges are outgoing and incoming and may lead to a misunderstanding of the whole biochemical network structure [22], [23], [42], [43]. The ratio of node-edge crossings is defined as the ratio of the number of node-edge crossings to the total number of node-edge combinations. Here, we set both the width and the height of node labels to 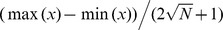, where max(*x*) and min(*x*) are the maximum and minimum values of *x* axis in the nodes, respectively.

#### Relative edge length

The relative edge length is defined as:



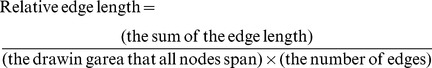



The relative edge length can indicate the efficiency of drawings. A small value of the relative edge length indicates the total edge length necessary for drawing the whole map is short, which indicates a high efficiency of drawings and would suggest well-balanced distributions of nodes as shown in **Figure S3**.

#### Connectivity F-measure

Generally adjacent and nonadjacent nodes should be closely and far located in geometry, respectively. To characterize the geometric performance of the layout, the idea of the F-measure is employed that is widely used in the field of information retrieval. The connectivity F-measure is defined as the weighted harmonic average of precision and recall [71]. Let *#P* be the number of elements in set *P*. The precision  for the *i-*th element circle 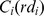 is defined by:



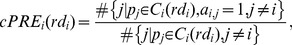



where 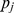 is the nodes and 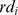 is the radius of 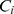. Next, the recall 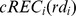 is defined as:



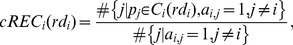



Roughly speaking, high precision favors a small 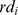 and high recall favors a large value of 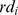; the optimal 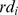 should be found in between them. The optimal radius 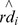
is chosen for each *i* that maximizes the following F-measure with weight factor 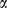. (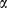 = 1/2 is used throughout our experiments.).



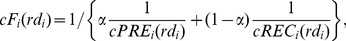



The measure to evaluate an embedded network layout, denoted as the connectivity F-measure, is defined by:



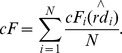



A large value of the connectivity F-measure shows that the adjacent and nonadjacent nodes are closely and far located with respect to each node, respectively. The connectivity F-measure is illustrated in **Figure S4**.

#### Functional F-measure

To identify biologically functional modules in the map, nodes with the same function should be located closely. The functional F-measure is defined that measures the degree to which the nodes with the same biological function are closely located and the nodes with different functions are far located, while the connectivity F-measure determines the degree to which the adjacent and nonadjacent nodes are closely and far located, respectively. The precision 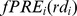 for the *i-*th geometric center element circle 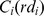 in a functional module 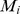 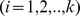 is defined by:



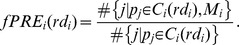



where 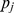 is the nodes and 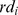 is the radius of 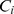. Next, the recall 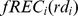 is defined by:



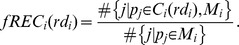



The optimal radius 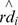 is chosen for each *i* that maximizes the following F-measure with weight factor 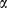. (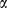 = 1/2 is used throughout our experiments.).



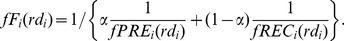



Since *k* is the number of functional modules, the measure to evaluate an embedded network layout, denoted as the functional F-measure, is defined by:



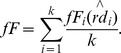



A large value of the functional F-measure shows that the nodes with the same function and with different functions are closely and far located in the map, respectively. The functional F-measure is illustrated in **Figure S5**.

### Implementation

The hybrid layout algorithm is implemented as a Windows Matlab (32bit) application software that consists of the input function for biochemical networks, the preprocessor with different layout algorithms, the pattern matching algorithm, and visualization program of resultant networks. MatlabBGL (http://www.stanford.edu/~dgleich/programs/matlab_bgl/) is used to compile the preprocessor algorithms from the Boost library into Matlab programs. The approximate pattern matching algorithm is written in C language. The visualization tool BNV2.0 is written in JAVA. These algorithms are called by the Matlab program. The test machine is the Intel Core2Duo (3.0 GHz) with memory 3.2 GBytes. CE [42], [43] uses the formatted networks by Cytoscape (http://www.cytoscape.org/) [14] to calculate the coordinates of the nodes, exporting the coordinates calculated.

### The graphical User Interface

The visualization tool (BNV2.0) was developed based on the JGraph tool of LucidDraw. JGraph (http://www.jgraph.com/jgraph.html) is an open source graph visualization library written in Java. BNV2.0 supports interactive operations on the network drawings such as moving nodes, zooming in/out, showing/hiding labels, and editing functions like redo/undo. BNV2.0 implements an additional function to search a node and modules, which highlights a target node by changing its frame color (**Figure S6**). To make easy use of BNV2.0 in the Matlab environment, GUI (**Figure S7**) was developed to provide an intuitive way to manipulate input network data and adjust the detailed parameters necessary for layout.

### Biochemical Network Maps

To demonstrate the feasibility of the hybrid algorithms, we applied them to 16 metabolic networks in KEGG [72] (**Table 2**), which are converted into the CADLIVE format by the CADLIVE Converter [73].

**Table 2:** Biochemical networks used by the proposed layout algorithms.

Number	Network	Nodes	Edges
1	Glycan Biosynthesis and Metabolite	54	61
2	Nucleotide Metabolism	160	236
3	Metabolism of Other Amino Acids	201	246
4	Energy Metabolism	214	323
5	Biosynthesis of Other Secondary Metabolism	261	288
6	Metabolism of Cofactors and Vitamines	370	430
7	Metabolism of Terpenoids and Polyketides	414	467
8	Lipid Metabolism	425	558
9	Carbohydrate Metabolism	540	845
10	Amino Acid Metabolism	613	812
11	Xenobiotics Biodegradation and Metabolism	652	768
12	3+10	781	1058
13	2+3+10	922	1290
14	1+7+8+11	1430	1759
15	2+3+4+5+6+9+10	2456	3483
16	All combinational networks in 1–11 networks	4198	5682

## Results and Discussion

### Biochemical Network Maps Drawn by the Hybrid Algorithms

**Figure 2:**
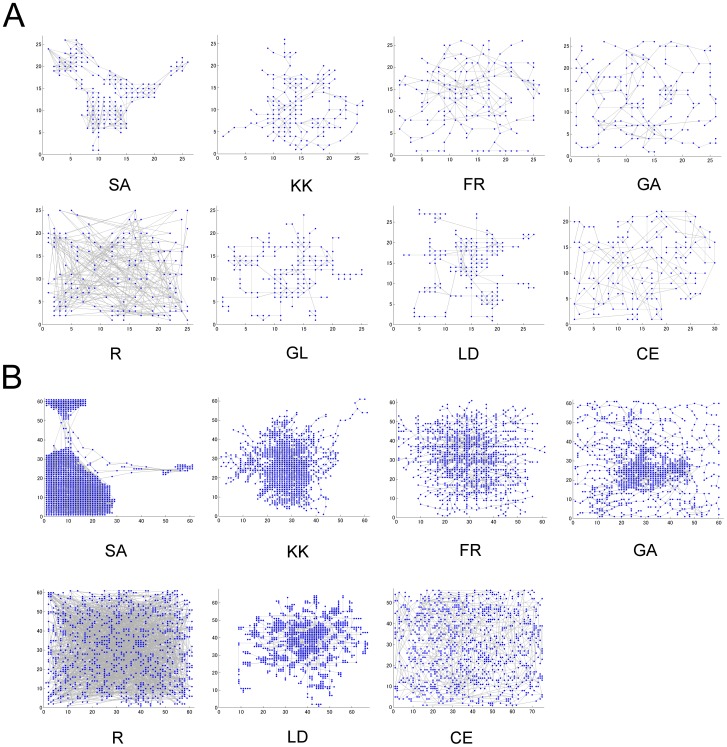
The metabolic network maps drawn by hybrid grid layout algorithms. A: The nucleotide metabolism network maps (Nodes: 160, Edges: 236). B: The amino acid and nucleotide metabolic network maps (Nodes: 922, Edges: 1290). Four types of the hybrid layout algorithms (SA, KK, FR, and GA) are used. Random layout (R), our grid layout (GL), LucidDraw (LD), and Cerebral (CE) are employed as reference algorithms. Here, their network maps are drawn by simple representation using circles and lines in the MATLAB program. GL in the network (B) does not build any network map due to the calculation complexity.

To investigate the performance of the hybrid layout algorithms with different preprocessors, SA, KK, FR and GA, they are applied to drawing of metabolic network maps with different sizes, as shown in **Figure 2**. As reference methods, a random layout (R), our original grid layout (GL), LucidDraw (LD), and Cerebral (CE) are employed. In the random layout algorithm, nodes are uniformly and randomly distributed in the given square area, as expected. On the other hand, the node distributions by the hybrid algorithms and reference ones show specific topological features, such as heterogeneous distributions and modular structures, respectively. Selection of the preprocessor algorithms, which calculate a coarse layout that gives a relative position to each node, affected the calculation speed and topology of the resultant layouts.

### Characterization of Hybrid Grid Layout Algorithms

**Figure 3:**
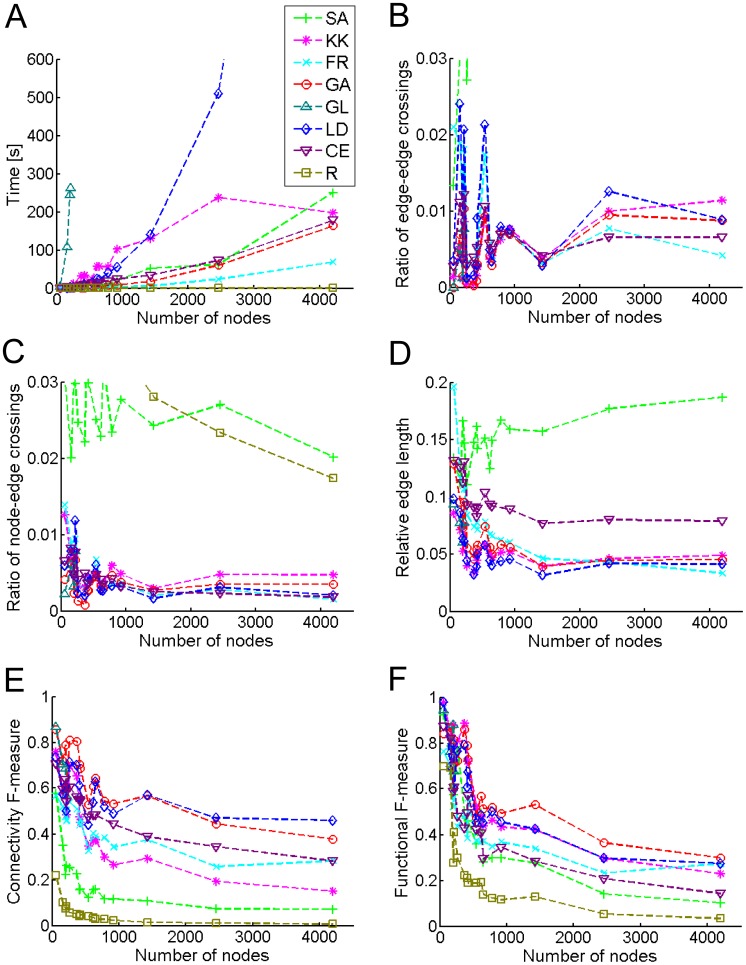
Characterization of hybrid layout algorithms. (A) calculation speed, (B) ratio of edge-edge crossings, (C) ratio of node-edge crossings, (D) relative edge length, (E) connectivity F-measure, and (F) functional F-measure. Four types of the hybrid layout algorithms (SA, KK, FR, and GA) are used. As reference methods, a random layout (R), our original grid layout (GL), LucidDraw (LD), and Cerebral (CE) are employed.

**Figure 4:**
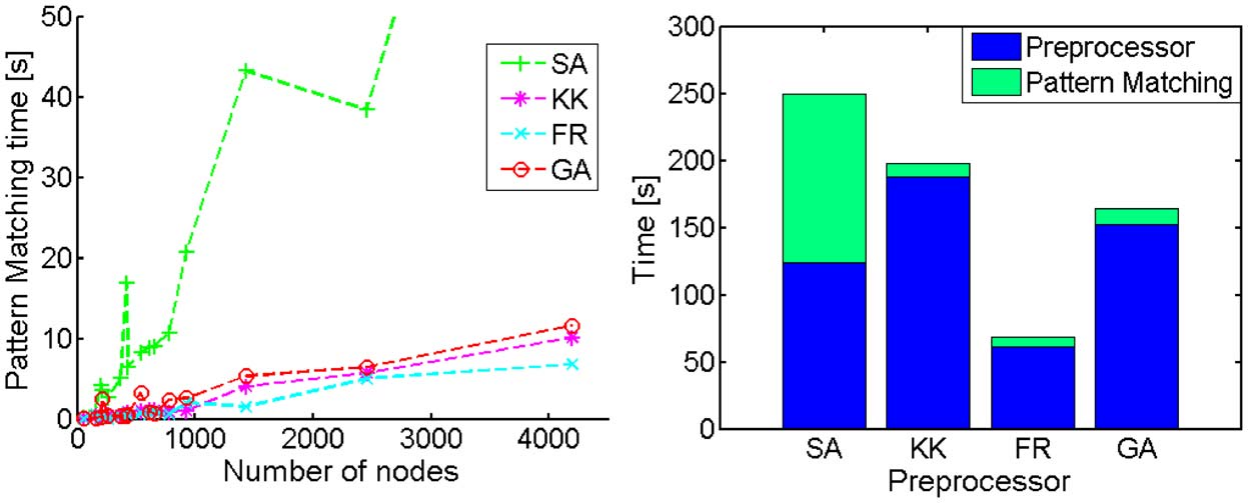
Computational time of hybrid grid layout algorithms. Left panel: The calculation time required for the pattern matching with respect to the number of nodes. Right panel: Time composition of the hybrid layouts calculating a network with 4198 nodes and 5682 edges (the network of number 16 in the Table2).

First, the calculation speed for the hybrid algorithms was evaluated as shown in **Figure 3A**. GL required lots of calculation time and could practically not calculate any map with more than several hundred nodes, while its topological performances were very good (**Figure 3B, C, D, E, F**). LD, an improved version of GL, calculated layouts faster than GL, but it was still slower than the hybrid layout algorithms. The two hybrid grid layout algorithms (FR and GA) were faster than CE or comparable to it, indicating that the hybrid algorithms can greatly increase the calculation speed. Although a random layout algorithm (R) was very fast, the geometric performance was very poor (**Figure 3B, C, D, E, F**). The approximate pattern matching for KK, FR, and GA was very fast, thus their calculation time depended on selection of the preprocessors (**Figure 4**). FR presented the highest calculation speed. On the other hand, SA was slowest and its pattern matching required a long time. It is because the spectral analysis provided highly heterogeneous node distributions (**Figure 2**), which make it hard to find vacant grid points. The speed of the pattern matching is suggested to be fast for the homogeneous node distributions generated by FR (**Figure 2**, **Figure 4**).

Second, the ratio of edge-edge crossings was characterized as shown in **Figure 3B**. R indicated the largest ratios (approximately 0.39) for all the networks (data not shown). The ratios of the edge-edge crossings by FR and GA were less than that by LD. The ratios by FR and CE were greatly reduced to a comparable level. FR could greatly reduce the ratio of edge-edge crossings. Third, the ratio of node-edge crossings was characterized as shown in **Figure 3C**. For most of the networks, the ratios of node-edge crossings by FR and GA were comparable to those by LD and CE.

Second, the ratio of edge-edge crossings was characterized as shown in **Figure 3B**. R indicated the largest ratios (approximately 0.39) for all the networks (data not shown). The ratios of the edge-edge crossings by FR and GA were less than that by LD. The ratios by FR and CE were greatly reduced to a comparable level. FR could greatly reduce the ratio of edge-edge crossings. Third, the ratio of node-edge crossings was characterized as shown in **Figure 3C**. For most of the networks, the ratios of node-edge crossings by FR and GA were comparable to those by LD and CE.

Fourth, the relative edge length was characterized as shown in **Figure 3D**. R indicated the largest length (approximately 0.52) for all the networks (data not shown). For most of the networks, the relative edge lengths by KK, FR, and GA were as short as that by LD, while they were less than that by CE. KK, FR and GA present a short relative edge length, showing well-balanced layouts.

**Figure 5:**
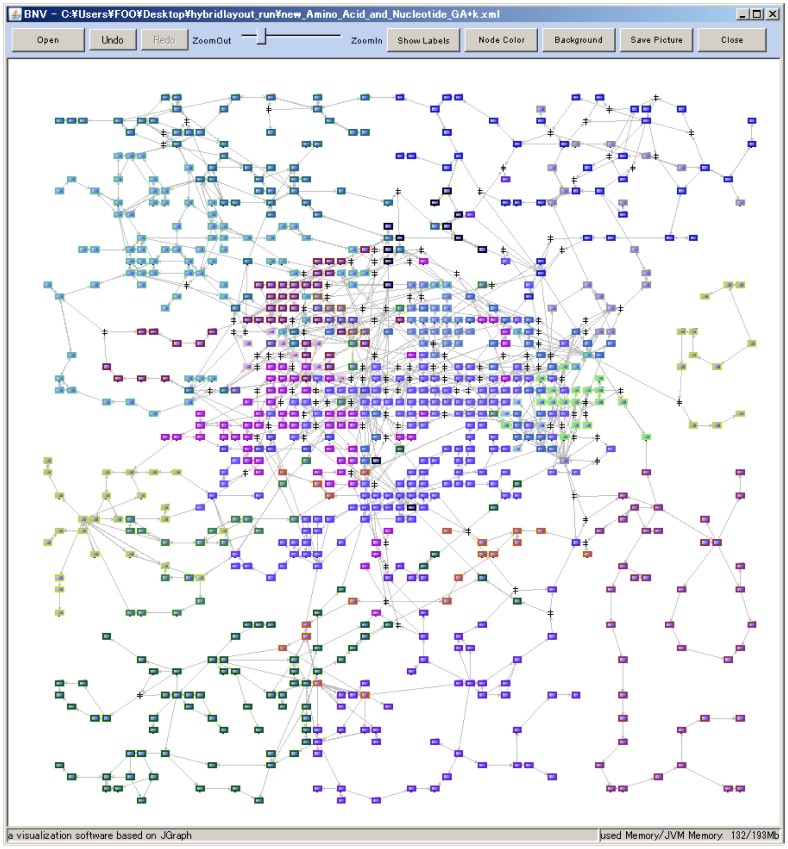
An amino acid and nucleotide metabolism network map (Nodes: 922, Edges: 1290) drawn by BNV2.0. The node coordinates are calculated by the hybrid layout algorithm (GA), which presents the best score (0.49) of the functional F-measure. There are 24 modules in the map, where the nodes in each module are marked in different colors.

Fifth, the connectivity F-measure was evaluated as shown in **Figure 3E**. R showed the lowest value for all the networks, as had been expected. LD presented the highest connectivity F-measure. For all the networks, the connectivity F-measures by FR and GA were comparable to it and higher than those by CE, respectively. Finally, the functional F-measure was evaluated in terms of biologically functional modules annotated by KEGG (Glycolysis, TCA cycle, Pentose phosphate, etc.), as shown in **Figure 3F**. For most of the networks, the functional F-measures by KK, FR and GA were higher than those by CE. GA presented the highest functional F-measure. To visually demonstrate how the drawn network maps by GA are related to biological functions, the amino acid and nucleotide metabolism network (the network of number 13 in the Table 2) was illustrated in **Figure 5**. The nodes are marked by BNV2.0 in different colors according to biological functions, clearly presenting biologically related cluster structures. It presents the highest score (0.49) for the functional F-measure.

In summary, selection of the preprocessor algorithms affected the calculation speed and topology of the resultant layouts. The calculation speeds of FR and GA are faster than other existing methods. FR and GA show high or comparable performances for the other topological measures compared with CE. GL and LD present high performances for some topological measures, but their calculation speeds are very slow.

### Time Complexity of the Hybrid Layout Algorithms

The time complexity of the pattern matching and preprocessor is estimated separately. When the entire area with *N* points (nodes) is divided by axis-parallel lines into subsections with at most *k* points, the number of stages which halves each subsection is no greater than 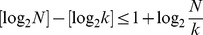. Since all *N* points are processed at every division stage, the time complexity to divide the entire area into subsections with at most *k* points is:



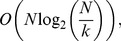



and equals to 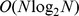 if *k* is independent of the number of nodes *N* or constant. Since finding the best pattern matching for *k* points for each subsection requires time proportional to 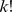 and the number of subsections is given by two to the power of the number of depths of division stages, the total time complexity for pattern matching is:



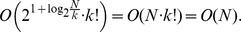



The time complexity for pattern matching of all *N* points is:



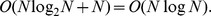



On the other hand, time complexity of FR is 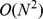. Therefore, the total time complexity of the hybrid layout with FR is provided by 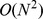.

When the division number is large, i.e., the number of points within each subsection (*k*) is small, the calculation speed for the pattern matching is greatly enhanced as shown in **Figure S2**. It is important to determine the division number so as to enhance the calculation speed without losing readability of graph layouts.

### Comparison with a Non-Grid Layout Algorithm

We focus on the grid layouts, while many scientists would be interested in the performance of fast, non-grid layout algorithms that avoid the overlapping of node labels. As well as the proposed hybrid layout algorithm, the Dwyer’s method consists of two processes: the preprocessor and the subsequent algorithm to avoid node-overlapping [45]. The Dwyer’s method employs a layout adjustment algorithm instead of pattern-matching algorithms. The layout adjustment by Dwyer et al. is known to be very fast. Its time complexity is 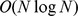 to produce a set of constraints necessary for non-overlapping. An exponential time is theoretically required to solve the constraint satisfaction problem. Actually the problem can quickly be solved when the number of overlapping nodes is not extremely high. We compared the hybrid layout method with the Dwyer’s method while using the same preprocessor algorithm (GA), as shown in **Text S1, Figure S8 and Figure S9**. The proposed hybrid layout algorithm was slower than the Dwyer’s method, but a second-order fast algorithm. It is practically feasible enough. The hybrid grid layout still takes an advantage in topological performances (short relative edge length, high functional F-measure, well-shaped outline), or orderly, well-balanced drawings.

### Conclusions

In order to enhance the understanding or traceability of biochemical networks it is practically important to avoid the overlapping of node labels and to arrange them in a geometrically aesthetic manner. Out of many algorithms, we focused on the grid layout algorithms [22]–[25], [42], [43], [56], because they arranged all the nodes to geometrically aesthetic grid points to provide orderly and balanced network map. The proposed hybrid grid layout algorithm consists of a widely-used, non-grid, fast layout (preprocessor) algorithm and an approximate pattern matching algorithm that redistributes the resultant preprocessed nodes on square grid points, while preserving the topological relationships among the nodes. To demonstrate the outstanding performance of the hybrid grid layout algorithm, it is compared with other existing grid layout methods (GL, LD, and CE) in terms of the calculation time, edge-edge and node-edge crossings, edge length, and connectivity and functional F-measure. The hybrid layout algorithms FR and GA not only enhance the calculation speed, but also improved geometric performances.

There can be many candidates of preprocessor algorithms, but the investigation of all candidates is not practical. Four typical algorithms (SA, KK, FR, and GA) are used to demonstrate the feasibility of the hybrid grid layouts. Use of latest algorithms [60]–[64] as the preprocessors would be a next task to investigate if they further improve the performance of the hybrid layout algorithms.

## Supporting Information

Figure S1 **A search order for finding the nearest vacant grid point.**
(PDF)

Figure S2 **Calculation time required for pattern matching by the depth-first recursive algorithm.**
(PDF)

Figure S3 **Illustration for the relative edge length.**
(PDF)

Figure S4 **Precision and Recall for calculating the connectivity F-measure.**
(PDF)

Figure S5 **Precision and Recall for calculating the functional F-measure.**
(PDF)

Figure S6 **The whole metabolic network map (Nodes: 4198, Edges: 5682) drawn by BNV2.0.**
(PDF)

Figure S7 **GUI for executing the hybrid layout algorithm.**
(PDF)

Figure S8 **Comparison of the layout performance between the node adjustment algorithm by Dwyer et al. and our pattern matching algorithm.**
(PDF)

Figure S9 **Node distributions in the network (Nodes: 922, Edges: 1290).**
(PDF)

Table S1 **Pattern matching algorithm.**
(PDF)

Table S2 **Search of the nearest, vacant grid point.**
(PDF)

Text S1 **Comparison with non-grid layout algorithms.**
(PDF)
