# Supplementary material for: Application of Approximate Pattern Matching in Two Dimensional Spaces to Grid Layout for Biochemical Network Maps
Source: PLoS One. 2012 Jun 5;7(6):e37739. doi: 10.1371/journal.pone.0037739 (PMC3368000; doi:10.1371/journal.pone.0037739)
Supplement: Figure S1 — A search order for finding the nearest vacant grid point. (PDF) [file pone.0037739.s001.pdf]

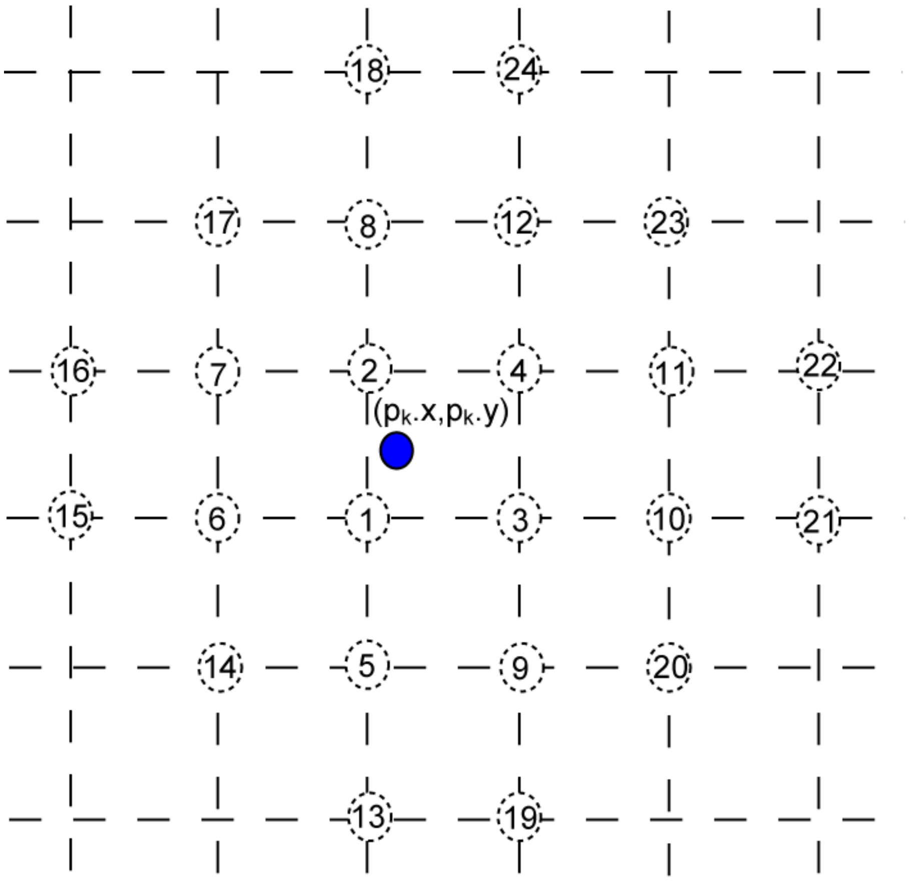

Figure S1. A search order for finding the nearest vacant grid point.

The blue circle is a node of p_k. The index number within each grid point indicates the search order for finding the nearest vacant grid point.
