# Supplementary material for: Application of Approximate Pattern Matching in Two Dimensional Spaces to Grid Layout for Biochemical Network Maps
Source: PLoS One. 2012 Jun 5;7(6):e37739. doi: 10.1371/journal.pone.0037739 (PMC3368000; doi:10.1371/journal.pone.0037739)
Supplement: Figure S2 — Calculation time required for pattern matching by the depth-first recursive algorithm. (PDF) [file pone.0037739.s002.pdf]

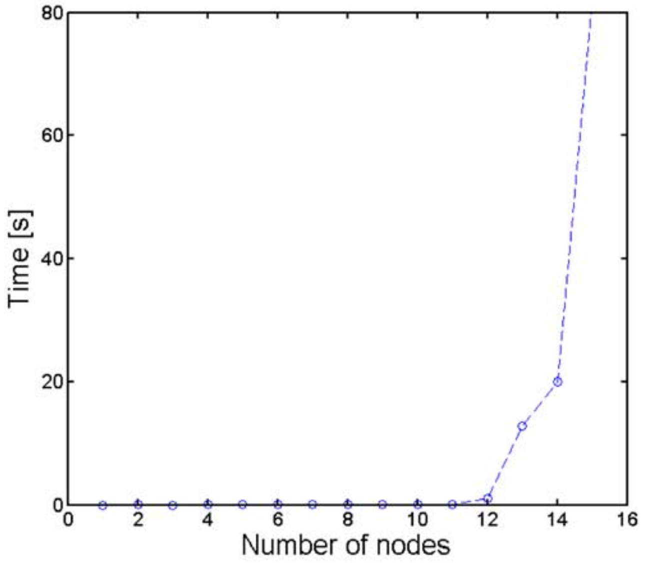

Figure S2. Calculation time required for pattern matching by the depth-first recursive algorithm.

Randomly laid-out nodes are redistributed to grid points by the pattern matching algorithm. The calculation time increased explosively with respect to the number of nodes (cut size) after 12 nodes.
