# Supplementary material for: Application of Approximate Pattern Matching in Two Dimensional Spaces to Grid Layout for Biochemical Network Maps
Source: PLoS One. 2012 Jun 5;7(6):e37739. doi: 10.1371/journal.pone.0037739 (PMC3368000; doi:10.1371/journal.pone.0037739)
Supplement: Figure S3 — Illustration for the relative edge length. (PDF) [file pone.0037739.s003.pdf]

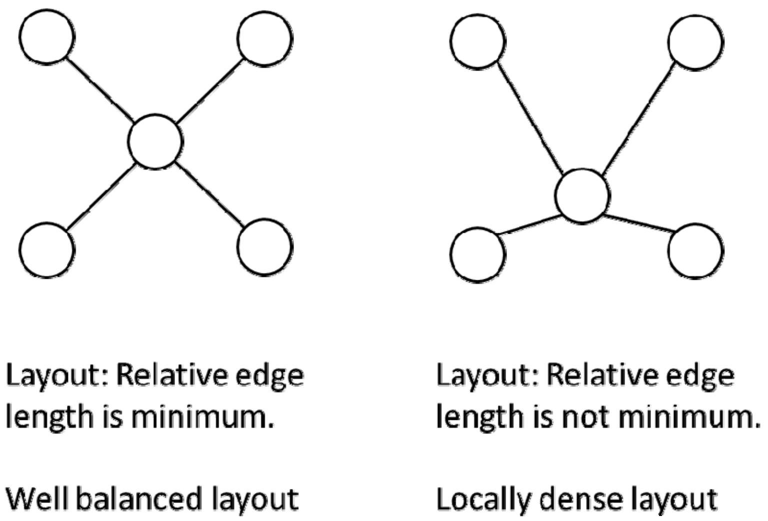

Figure S3. Illustration for the relative edge length.

The minimum relative edge length provides a proportional or balanced layout.
