# Supplementary material for: Application of Approximate Pattern Matching in Two Dimensional Spaces to Grid Layout for Biochemical Network Maps
Source: PLoS One. 2012 Jun 5;7(6):e37739. doi: 10.1371/journal.pone.0037739 (PMC3368000; doi:10.1371/journal.pone.0037739)
Supplement: Figure S4 — Precision and Recall for calculating the connectivity F-measure. (PDF) [file pone.0037739.s004.pdf]

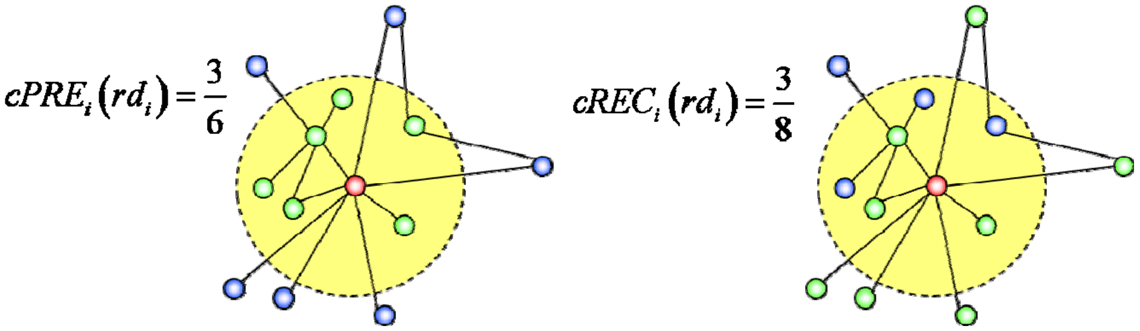

Figure S4. Precision and Recall for calculating the connectivity F-measure.

This figure illustrates how to calculate the connectivity F-measure. Red node *i* is the center of the yellow circle (module) with a radius of rd_i. In the left panel, six nodes (green) are located within the yellow circle, and node *i* has three adjacent nodes out of the six nodes. The precision value is given as $cPRE_i(rd_i) = \frac{3}{6}$. In the right panel, node *i* has eight adjacent nodes (green). Out of the eight nodes, three nodes are adjacent to node *i* within the yellow circle. The recall value is given as $cREC_i(rd_i) = \frac{3}{8}$.
