# Supplementary material for: Application of Approximate Pattern Matching in Two Dimensional Spaces to Grid Layout for Biochemical Network Maps
Source: PLoS One. 2012 Jun 5;7(6):e37739. doi: 10.1371/journal.pone.0037739 (PMC3368000; doi:10.1371/journal.pone.0037739)
Supplement: Figure S5 — Precision and Recall for calculating the functional F-measure. (PDF) [file pone.0037739.s005.pdf]

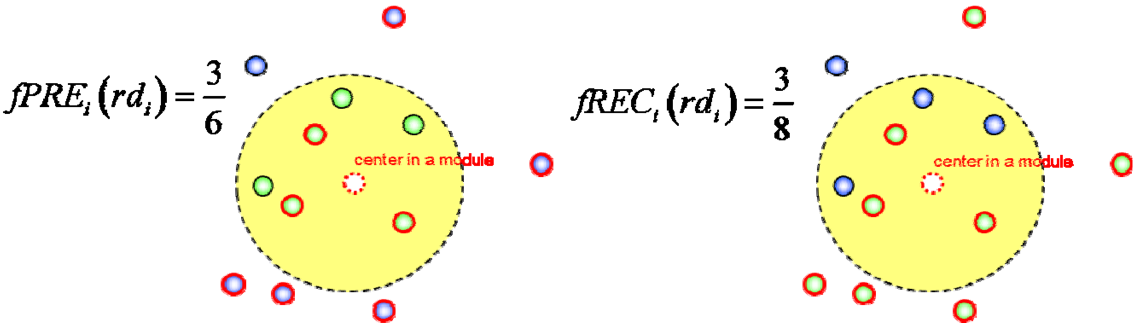

Figure S5. Precision and Recall for calculating the functional F-measure.

This figure illustrates how to calculate the functional F-measure. Small red dotted circle *i* is the center of the yellow circle (module) with a radius of rd_i. In the left panel, six nodes (green) are located within the yellow circle, and three nodes has the same function as node *i* out of the six nodes. The precision value is given as $fPRE_i(rd_i)=\frac{3}{6}$. In the right panel, eight nodes (green) has the same function as node *i*. Out of the eight nodes, three nodes are located within the yellow circle. The recall value is given as $fREC_i(rd_i)=\frac{3}{8}$.
