# Supplementary material for: Application of Approximate Pattern Matching in Two Dimensional Spaces to Grid Layout for Biochemical Network Maps
Source: PLoS One. 2012 Jun 5;7(6):e37739. doi: 10.1371/journal.pone.0037739 (PMC3368000; doi:10.1371/journal.pone.0037739)
Supplement: Figure S6 — The whole metabolic network map (Nodes: 4198, Edges: 5682) drawn by BNV2.0. (PDF) [file pone.0037739.s006.pdf]

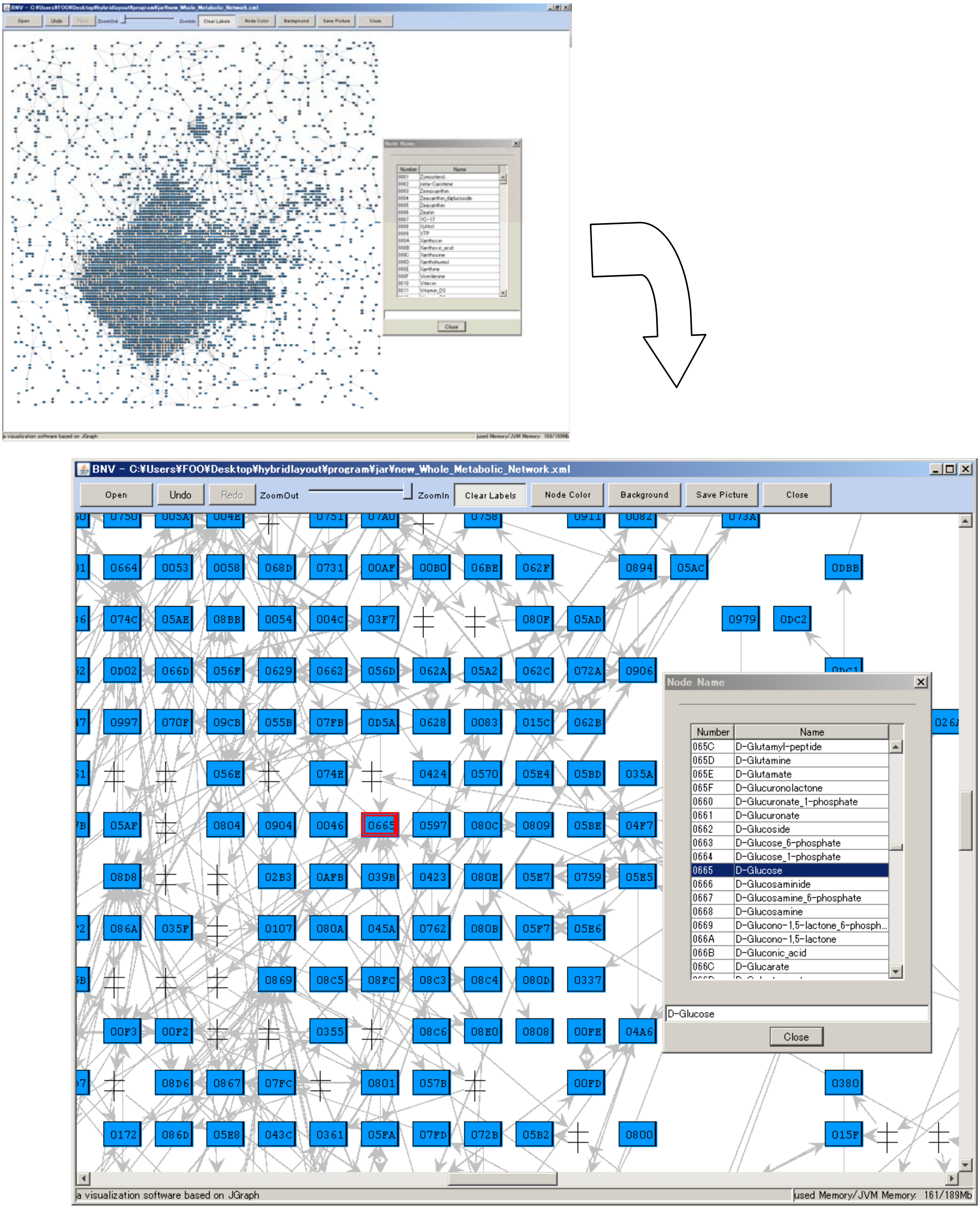

Figure S6. The whole metabolic network map (Nodes: 4198, Edges: 5682) drawn by BNV2.0.

The node coordinates were calculated by the hybrid layout algorithm (GA). A specific node (D-Glucose) can be readily found in the map, because it is highlighted in red.
