# Supplementary material for: Application of Approximate Pattern Matching in Two Dimensional Spaces to Grid Layout for Biochemical Network Maps
Source: PLoS One. 2012 Jun 5;7(6):e37739. doi: 10.1371/journal.pone.0037739 (PMC3368000; doi:10.1371/journal.pone.0037739)
Supplement: Figure S7 — GUI for executing the hybrid layout algorithm. (PDF) [file pone.0037739.s007.pdf]

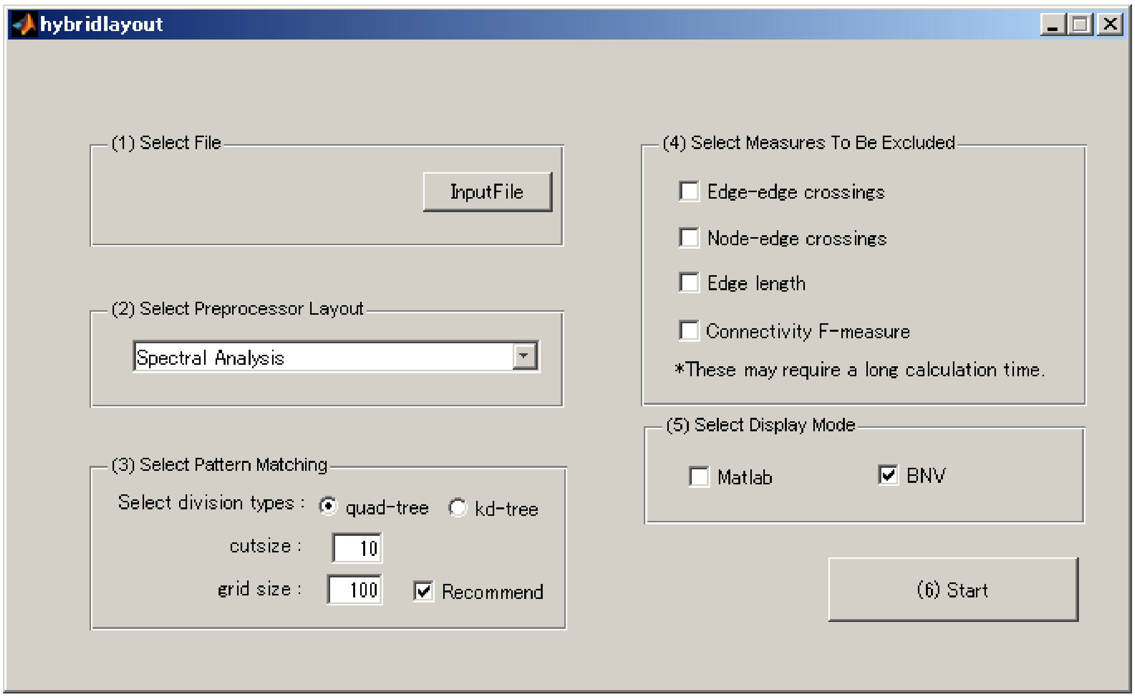

Figure S7. GUI for executing the hybrid layout algorithm.

User can import network data and set the parameters through this GUI. Details are described in the Instruction file in hybridlayout.zip (http://www.cadlive.jp/hybridlayout/hybridlayout.zip).
