# Supplementary material for: Application of Approximate Pattern Matching in Two Dimensional Spaces to Grid Layout for Biochemical Network Maps
Source: PLoS One. 2012 Jun 5;7(6):e37739. doi: 10.1371/journal.pone.0037739 (PMC3368000; doi:10.1371/journal.pone.0037739)
Supplement: Figure S8 — Comparison of the layout performance between the node adjustment algorithm by Dwyer et al. and our pattern matching algorithm. (PDF) [file pone.0037739.s008.pdf]

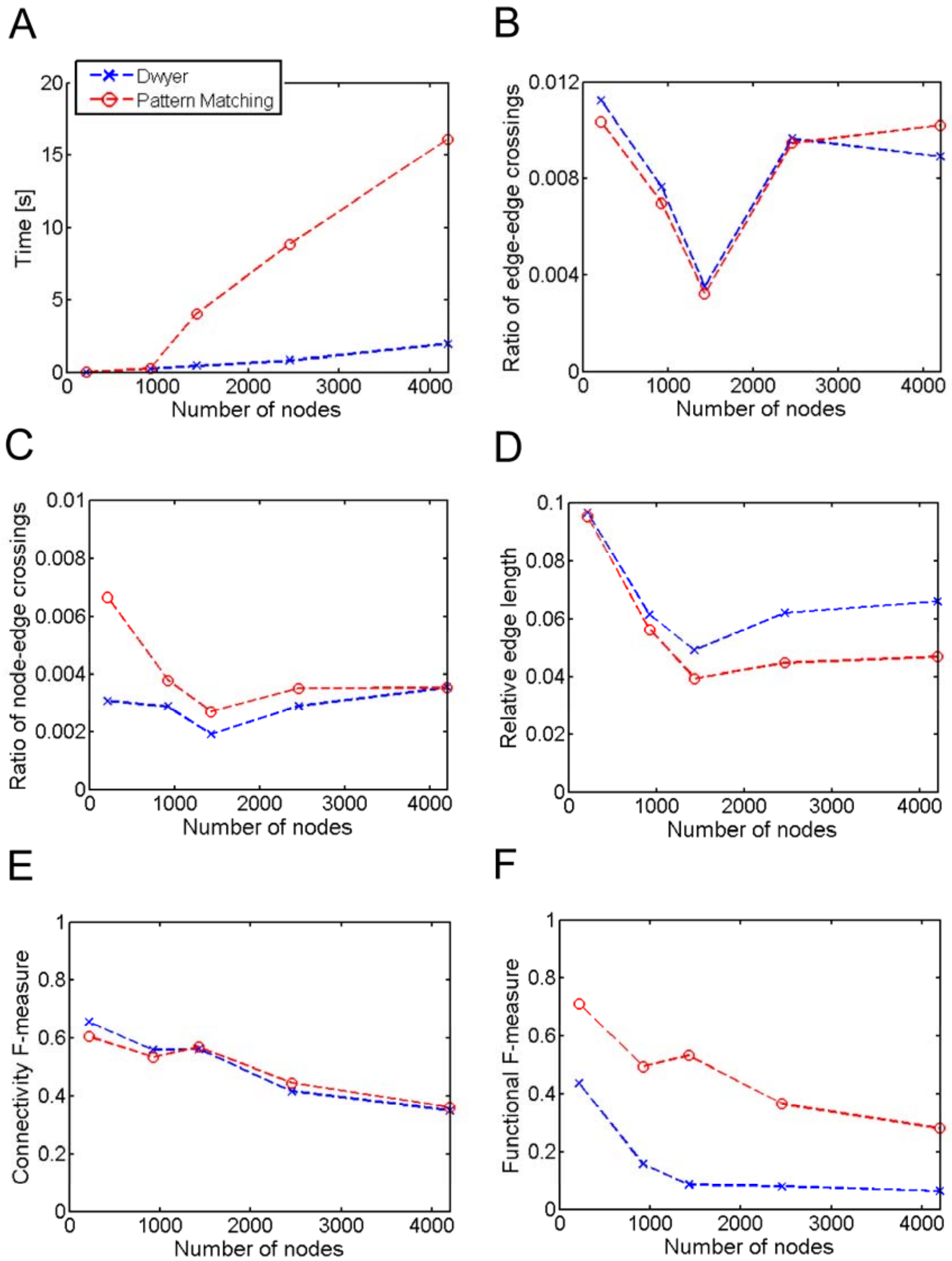

Figure S8. Comparison of the layout performance between the node adjustment algorithm by Dwyer et al. and our pattern matching algorithm.

(A) calculation speed in the Dwyer's method and our pattern matching algorithm, (B) ratio of edge-edge crossings, (C) ratio of node-edge crossings, (D) relative edge length, (E) connectivity F-measure, and (F) functional F-measure.
