# Supplementary material for: Application of Approximate Pattern Matching in Two Dimensional Spaces to Grid Layout for Biochemical Network Maps
Source: PLoS One. 2012 Jun 5;7(6):e37739. doi: 10.1371/journal.pone.0037739 (PMC3368000; doi:10.1371/journal.pone.0037739)
Supplement: Figure S9 — Node distributions in the network (Nodes: 922, Edges: 1290). (PDF) [file pone.0037739.s009.pdf]

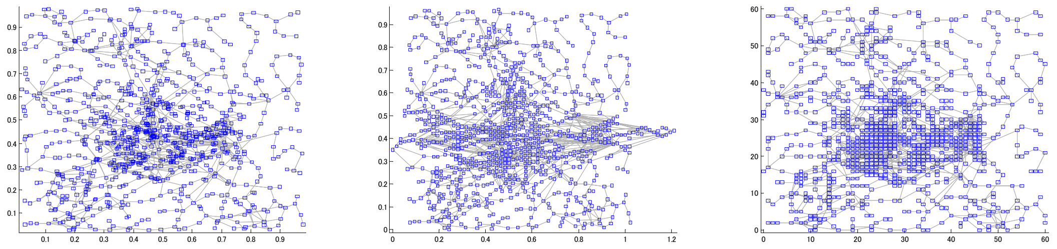

Figure S9. Node distributions in the network (Nodes: 922, Edges: 1290).

Left: Laid-out node distribution by the GA preprocessor. Middle: Node distribution by the Dwyer's method. Right: Node distribution by the pattern matching.
