# Supplementary material for: Application of Approximate Pattern Matching in Two Dimensional Spaces to Grid Layout for Biochemical Network Maps
Source: PLoS One. 2012 Jun 5;7(6):e37739. doi: 10.1371/journal.pone.0037739 (PMC3368000; doi:10.1371/journal.pone.0037739)
Supplement: Table S1 — Pattern matching algorithm. (PDF) [file pone.0037739.s010.pdf]

Table S1. Pattern matching algorithm.

global variables

N,
D[1:N], minimumDistance,
P, G, tempG[1:N]

Search(*i*) //

if ($i == N$)
    if (*minimumDistance* > $D(N)$)
        minimumDistance= $D(N)$
        $G = tempG(N)$
    endif
elseif ($i < N$)
    for k = i + 1: N
        NodeMatching(*k*)
            if (*minimumDistance* < $D(k)$)
                break
            else
                Search(*i*+1)
            endif
    endfor
endif

NodeMatching(*k*) //

j = 1 //search order index for grid points
do while (the nearest vacant grid point is not found)
    if ($gs_k(j)$ is not vacant) OR ($gs_k(j)$ is out of the setting square)
        j=j+1 // next grid point
    else
        $g_k = gs_k(j)$
        p_u^{update} <- k+1 to N-th nodes of P are parallelly moved together with the movement of p_k^{update} to g_k .
        $d_k = ds_k(j)$
    endif
enddo
$tempG(k) = \{tempg \mid tempg_i = g_i \ (i = 1, 2, ..., k),\ tempg_u = p_u^{update} (u = k+1, ..., N)\}$
$D(k) = D(k\text{-}1) + d_k$

i is the number of the matched nodes. i starts with zero. Before pattern matching, $tempG = P$.
