# Supplementary material for: Application of Approximate Pattern Matching in Two Dimensional Spaces to Grid Layout for Biochemical Network Maps
Source: PLoS One. 2012 Jun 5;7(6):e37739. doi: 10.1371/journal.pone.0037739 (PMC3368000; doi:10.1371/journal.pone.0037739)
Supplement: Table S2 — Search of the nearest, vacant grid point. (PDF) [file pone.0037739.s011.pdf]

Table S2. Search of the nearest, vacant grid point.

When $p_k.x - n_k.x < 0.5 \cap p_k.y - n_k.y < 0.5 \cap \frac{p_k.y - n_k.y}{p_k.x - n_k.y} \geq 1$, the *k*-th grid point candidates $gs_k(j)$ are sorted in the ascending order of their Manhattan distances.

The search order of the nearest, vacant grid point	Coordinate of the grid point candidate	Distance from node *k* to the grid point candidate
1	$gs_k(1) = (n_k.x, n_k.y)$	$ds_k(1) = \| p_k.x - n_k.x \| + \| p_k.y - n_k.y \|$
2	$gs_k(2) = (n_k.x, n_k.y + 1)$	$ds_k(2) = \| p_k.x - n_k.x \| + \| 1 - (p_k.y - n_k.y) \|$
3	$gs_k(3) = (n_k.x + 1, n_k.y)$	$ds_k(3) = \| 1 - (p_k.x - n_k.x) \| + \| p_k.y - n_k.y \|$
4	$gs_k(4) = (n_k.x + 1, n_k.y + 1)$	$ds_k(4) = \| 1 - (p_k.x - n_k.x) \| + \| 1 - (p_k.y - n_k.y) \|$
5	$gs_k(5) = (n_k.x - 1, n_k.y)$	$ds_k(5) = ds_k(1) + 1$
6	$gs_k(6) = (n_k.x, n_k.y - 1)$	$ds_k(6) = ds_k(1) + 1$
7	$gs_i(7) = (n_i.x - 1, n_i.y + 1)$	$ds_i(7) = ds_i(2) + 1$
8	$gs_k(8) = (n_k.x, n_k.y + 2)$	$ds_k(8) = ds_k(2) + 1$
9	….	….
