# Supplementary material for: Application of Approximate Pattern Matching in Two Dimensional Spaces to Grid Layout for Biochemical Network Maps
Source: PLoS One. 2012 Jun 5;7(6):e37739. doi: 10.1371/journal.pone.0037739 (PMC3368000; doi:10.1371/journal.pone.0037739)
Supplement: Text S1 — Comparison with non-grid layout algorithms. (PDF) [file pone.0037739.s012.pdf]

Text S1. Comparison with non-grid layout algorithms

We have compared our pattern matching algorithm with the layout adjustment algorithm employed by the Dwyer's method, for the input graphs preprocessed by the same preprocessor algorithm, GA. The size of node labels is supposed to be less than fixed grid width when we apply the hybrid layout method. As the grid width, we have chosen the length that makes $2\sqrt{N}$ grid lines for each side of the axis-parallel bounding box of nodes. Since the Dwyer's method allows an arbitrary size of the label for each node, we set both the width and the height of node labels to $(\max(x)-\min(x))/(2\sqrt{N}+1)$, where max($x$) and min($x$) are the maximum and minimum values of x axis in the nodes, respectively. We employed the libvpsc library (https://github.com/mjwybrow/adaptagrams) for the Dwyer's method.

The Dwyer's method, which makes a set of constraint rules in $O(N \log N)$ time then solves the rules, runs faster than our hybrid layout method that does $O(N \log N)$ -time divide-conquer then pattern matching (**Figure S8A**). Evaluations of obtained layouts by the edge-edge crossings and connectivity F-measures seem to be almost the same (**Figure S8BE**). The node-edge crossings (nodes on the edges) in the Dwyer‘s method are smaller than our hybrid layout method (**Figure S8C**). It is straightforward, as a graph layout scheme, that the grid graph drawing places many nodes on the same line and thus the nodes are on the edges, while the Dwyer‘s method does not employ such constraints.

The relative edge length by our pattern matching algorithm is smaller than that by the Dwyer's method, which suggests that the pattern matching provides well-balanced drawings (**Figure S8D**). It should be noticed that the functional F-measures by our pattern matching algorithm are higher than those by the Dwyer's method (**Figure S8F**).

The Dwyer's method redistributes merely the overlapping nodes by enforcing non-overlap in single dimension (x or y direction), while fixing the non-overlapping nodes. It hardly considers changes in the topological relationship between the redistributed nodes and the fixed nodes, resulting in some deterioration of topological relationships among the laid-out nodes by the preprocessor. Some regions of the map are sticking out, while the map generated by the pattern matching is well confined within the square (**Figure S9, middle, right**). The pattern matching algorithm tends to conserve the topological node distribution (**Figure S9 right**), because all the non-matched nodes move in parallel together with the matched node (See Method). This would

be the reason why the pattern-matching algorithm increases the functional F-measure.

In conclusion, the proposed hybrid algorithm is slower than the Dwyer’s method. However, it is efficient enough in practice. Moreover, the hybrid grid layout still takes an advantage of topological performances (short relative edge length, high functional F-measure, well-shaped outline), in drawings, or informally, of drawing well-ordered and well-balanced graphs.
